# ESKAPE pathogens and their antimicrobial resistance patterns at the university teaching hospital of Kigali, Rwanda: a five -year analysis (2020–2024)

**DOI:** 10.3389/fpubh.2026.1783011

**Published:** 2026-04-23

**Authors:** Marthe Uwamariya, Theophile Dushimirimana, Denyse Akayezu, Angelique Mukanyandwi, Francois Regis Musana Muhire, Masceline Jenipher Mutsaka-Makuvaza, Lisine Tuyisenge, Martin Nyundo, Tharcisse Mpunga, Jean Bosco Munyemana

**Affiliations:** 1 Planning, Monitoring, and Evaluation Unit, University Teaching Hospital of Kigali, Kigali, Rwanda; 2 Department of Surgery, University Teaching Hospital of Kigali, Kigali, Rwanda; 3 Department of Pathology, University Teaching Hospital of Kigali, Kigali, Rwanda; 4 Department of Microbiology and Parasitology, School of Medicine and Pharmacy, College of Medicine and Health Sciences, University of Rwanda, Kigali, Rwanda; 5 Department of Pediatrics, University Teaching Hospital of Kigali, Kigali, Rwanda; 6 University Teaching Hospital of Kigali, Kigali, Rwanda

**Keywords:** antimicrobial resistance, CHUK, ESBL, ESKAPE pathogens, MDR

## Abstract

**Background:**

Antimicrobial resistance (AMR) in *Enterococcus faecium, Staphylococcus aureus, Klebsiella pneumoniae, Acinetobacter baumannii, Pseudomonas aeruginosa*, and *Enterobacter spp*., known as ESKAPE pathogens, continue to pose a health threat globally. In Rwanda, data on long-term trends and resistance profiles among these pathogens remain limited. This study has assessed the temporal trends in the distribution and AMR patterns of ESKAPE pathogens at the University Teaching Hospital of Kigali (CHUK) from 2020 to 2024.

**Methodology:**

A retrospective laboratory-based surveillance study was conducted using microbiology laboratory data. All culture-positive clinical specimens processed over the five years were included. Descriptive analysis was performed to assess pathogens' distribution, resistance rates, phenotypic resistance patterns, and temporal trends according to antibiotic categories.

**Results:**

A total of 3,778 culture-positive isolates were analyzed. *Klebsiella pneumoniae* was the most prevalent (43.8%), followed by *Staphylococcus aureus* (26.4%) and *Acinetobacter baumannii* (15.1%). The overall AMR rate was 56.5%, with the highest resistance observed in *Enterococcus spp*. (74.6%), *Acinetobacter baumannii* (69.9%), and *Enterobacter spp*. (64.4%). Resistance to access antibiotics increased from 54.7 % in 2020 to 83.3 % in 2024, while resistance to watch antibiotics rose from 42.5 % to 57.2%. ESKAPE Pathogens exhibited a comparatively lower resistance rate to the Reserve antibiotics, including polymyxin B and ceftazidime-avibactam. Phenotypic resistance analysis identified extended-spectrum β-lactamase-producing *Enterobacterales* (32.9 %), methicillin-resistant *Staphylococcus aureus* (31.1%), carbapenem-resistant *Enterobacterales* (15.6%), and vancomycin-resistant E*nterococci* (5%).

**Conclusion:**

This study demonstrates a high and increasing burden of multidrug-resistant ESKAPE pathogens, characterized by escalating resistance to first-line and second-line antibiotics and early indications of emerging resistance to last-resort antimicrobials. These findings highlight the urgent need to strengthen antimicrobial stewardship, infection prevention, and control measures.

## Introduction

Antimicrobial resistance (AMR) is a growing global public health emergency, associated with increased morbidity, mortality, and substantial healthcare costs (1). In 2024, an estimated 4.95 million deaths were associated with AMR, while 1.27 million of these deaths were directly attributable to AMR, and the projections suggest an increase in the annual death toll of 10 million people by 2050 if nothing is done (1, 2). Six leading pathogens responsible for this AMR-associated deaths were Escherichia *coli, Staphylococcus aureus, Klebsiella pneumoniae (K. pneumoniae), Streptococcus pneumoniae (S. pneumoniae), Acinetobacter baumannii (A. baumannii), and Pseudomonas aeruginosa (P. aeruginosa)*. Four of them (*S. aureus, K. pneumoniae, A. baumannii*, and *P. aeruginosa*) belong to the World Health Organization (WHO) priority pathogen known as ESKAPE pathogen (*Enterococcus, S. aureus, K. pneumoniae, A. baumannii, P. aeruginosa*, and *Enterobacter)* (3).

The ESKAPE pathogens are particularly important because they are capable of “escaping” the effects of many antimicrobial agents and are frequently implicated in hospital-acquired infections. These pathogens employ several mechanisms to resist to antibiotics, including hydrolytic enzyme production such as β-lactamase that inactivates β-lactam antibiotics; modification of antibiotic targets, which reduces drug binding; reduced membrane permeability, limiting antibiotic entry; and active efflux pumps that expel antibiotics from bacterial cells. Additionally, many ESKAPE pathogens acquire resistance genes through horizontal gene transfer, including plasmids and transposons, which accelerates the spread of multidrug resistance within healthcare settings (4).

In lower and middle-income countries (LMICs), hospital-acquired infections caused by ESKAPE pathogens exhibit resistance rates two to three times higher than those reported in high-income countries. Previous reports highlight that the resistance exceeds 50% among carbapenem-resistant *A. baumannii* and *K. pneumoniae* (5). Among LMICs, Africa bears a higher burden of AMR, largely due to inadequate diagnostic capacity, weak infection prevention and control (IPC) systems, and inappropriate antibiotic use (6).

To align with the ongoing global effort to tackle AMR through a multidisciplinary One Health approach (7); Rwanda has recognized AMR as a national priority and developed a national One Health Strategic Plan, which emphasizes strengthening antimicrobial surveillance, stewardship, and laboratory capacity to mitigate the AMR threat (8). A recent study has reported alarmingly high resistance rates among clinical isolates, with *S. aureus, K. pneumoniae*, and *A. baumannii* being among the most frequently identified pathogens with such resistance phenotypes (9). A study on antimicrobial susceptibility patterns of bloodstream infections at the University Teaching Hospital of Kigali (Centre Hospitalier Universitaire de Kigali:CHUK) reported a 77.1% prevalence of multidrug-resistant (MDR) organisms, with K. *pneumoniae* and *S. aureus* among the most frequently isolated organisms (10).

Moreover, another previous report has demonstrated a high rate of extended-spectrum β-lactamase (ESBL) producers among *E. coli* and *Klebsiella* isolates, and high resistance to oxacillin among *S. aureus* isolates (11). Despite these studies, systematic and long-term AMR surveillance remains limited, particularly for ESKAPE pathogens, limiting optimization of tailor-made empirical therapy, AMS interventions, and infection prevention strategies in Rwanda. Therefore, this study has characterized temporal trends in the distribution and antimicrobial resistance patterns of ESKAPE pathogens at CHUK over five years, to inform national AMR surveillance and control strategies.

## Materials and methods

### Study design and setting

This was a retrospective, laboratory-based surveillance study conducted at CHUK, a public tertiary referral and teaching hospital with around 500 beds serving patients from across Rwanda. The study analyzed bacteriological data generated by the microbiology laboratory over five years, from 1st January 2020 to 31st December 2024.

### Study population

The study population comprised all bacterial isolates obtained from eight key departments at CHUK, including Outpatient Department (OPD), Neonatology, Pediatrics, Internal Medicine (IM), Surgery, Intensive Care Unit (ICU), Accident and Emergency (A&E), and Obstetrics and Gynecology (OB-GYN).

### Sample size and sampling methods

The study employed a complete enumeration approach. All the culture-positive specimens processed at the CHUK microbiology laboratory during the study period were included. Overall, 3,778 clinical samples that yielded bacterial growth were included in the analysis. Specimens from patients of all age groups, including neonates, were included. Duplicates were removed before formal data analysis. All confirmed ESKAPE pathogen isolates recovered from blood, urine, pus, wound swabs, pleural fluid, tracheal aspirates and other sterile body fluids were analyzed. However, bacteria other than ESKAPE and isolates classified as contaminants based on laboratory criteria were excluded from the analysis.

### Laboratory isolation and identification of bacteria

All clinical specimens, including urine, blood, pus, swabs, and body fluids, were processed according to the standard operating procedures (SOPs) of the CHUK microbiology laboratory. Briefly, urine samples were examined microscopically and cultured on cysteine lactose electrolyte-deficient (CLED) agar (Oxoid, Thermo Fisher Scientific). Significant bacteriuria was defined as growth of ≥ 10^5^ colony-forming units (CFU)/mL in accordance with laboratory SOPs. This cutoff assisted in distinguishing true infection from contamination or colonization. Subsequently, species identification and antimicrobial susceptibility testing were performed. Blood culture bottles were incubated at 37°C using the automated BACTEC system (BD, Franklin Lakes, NJ, USA). Culture-positive bottles were subculture on sheep blood agar (SBA) (Oxoid, Thermo Fisher Scientific, UK) and incubated overnight at 37°C, in a 5% CO_2_ incubator. Other clinical samples were cultured on blood agar and MacConkey agar at the same time.

Bacterial identification was performed using standard microbiological techniques, including Gram staining, colony morphology assessment and conventional biochemical tests. Gram-negative isolates were identified using conventional biochemical tests including triple sugar iron (TSI), motility- indole-urease (MIU), oxidase testing, citrate utilization and lactose fermentation on MacConkey agar (Oxoid, Thermo Fisher Scientific, UK). *Pseudomonas aeruginosa* and *Acinetobacter* species identification was further based on sugar fermentation profiles, and characteristic growth features. Gram-positive isolates were identified based on Gram stain morphology, hemolysis pattern on blood agar, catalase and coagulase testing, and esculin hydrolysis for *Enterococcus spp*.

### Antimicrobial susceptibility testing

Antimicrobial susceptibility testing was performed using the Kirby-Bauer disk diffusion method on Mueller-Hinton (MH) agar, following Clinical and Laboratory Standards Institute (CLSI) M100 guidelines version 2021, 2022, 2023, and 2024, as the study involved microbiology culture data from 2020 to 2024. The turbidity of 0.5 McFarland inoculum was prepared from pure bacterial colonies and uniformly inoculated onto MH agar plates before disk placement. A panel of antibiotics commonly used in clinical practice is listed in Supplementary Table 1. Antibiotic selection for each isolate was guided by organism identity and routine laboratory protocols.

After disk placement, the plates were incubated at 37°C for 18–24 h. Inhibition zone diameters were measured and interpreted as susceptible, intermediate, or resistant based on CLSI breakpoints. ESBL production was determined according to CLSI-recommended phenotypic methods. CRE were defined as E. *coli* and *Klebsiella* species isolates resistant to meropenem or imipenem. MRSA was identified based on cefoxitin resistance.

### Quality control

To ensure accuracy and reliability of laboratory testing, quality control was performed throughout the process, following the internal SOPs using reference strains from the American Type Culture Collection (ATCC), including *Escherichia coli* ATCC 25922, *Klebsiella pneumoniae* ATCC 13883, and *Staphylococcus aureus* ATCC 25923 (Thermo Scientific, Lenexa, KS, USA).

### Data analysis

Microbiology laboratory data were extracted and entered into Microsoft Excel. Data was subsequently analyzed using R software, version 4.4.2 (2024-10-31 ucrt). Descriptive statistics were used to summarize demographic characteristics, specimen types, and pathogens distribution. Categorical variables were presented as frequencies and percentages. Differences in pathogen distribution and antimicrobial resistance proportions across years, specimen types, and hospital departments were assessed using the Chi-square test. Fisher's exact test was used when expected cell counts were less than five. A *p*-value of < 0.05 was considered statistically significant. To prevent duplicate counting, only the first isolate per patient per organism and resistance profile was included in the analysis. Antimicrobial resistance rates were calculated as the proportion of resistant isolates among the total number of isolates tested for each pathogen and antibiotic. Temporal trends in antimicrobial resistance over the five years were assessed descriptively by comparing annual resistance proportions, including analyses based on the World Health Organization (WHO) AWaRe (Access, Watch, Reserve) classification of antibiotics.

### Ethics approval and consent to participate

Ethical approval was obtained from the Institutional Review Board of the CHUK, under the reference number: EC/CHUK/171/2024. Informed consent was waived due to the retrospective nature of the study. To maintain patient confidentiality, all personal identifiers were removed, and data on the collection sheets were anonymized. The data were then stored in a password-protected computer database.

## Results

### Demographics characteristics

Overall, 3,778 patients with culture-positive specimens were included in the analysis. Among them, 2,027 (53.7%) were males, while 1,751 (46.4%) were females. Most patients were in the reproductive age group (19–45 years; *n* = 1,351; 35.8%), followed by old adults (>60 years; *n* = 702; 18.6%) and children under 5 years (*n* = 656; 17.4%). Most cases were from the Pediatrics department (*n* = 1,120; 29.7%), followed by the ICU (*n* = 614; 16.3%), and IM (*n* = 562; 14.9%) (Table 1).

**Table 1:** Demographic characteristics of patients with positive bacterial cultures.

	Characteristics	Frequency	%
Age categories	Neonates (0–28 days)	158	4.2
	Under five (1–59 months)	656	17.4
	Pediatrics (5–18 years)	467	12.4
	Reproductive age (19–45 years)	1,351	35.8
	Adults (46–60 years)	444	11.8
	Old adult (>60 years)	702	18.6
Gender	Female	1,751	46.4
	Male	2,027	53.7
Department	Pediatrics	1,120	29.7
	Intensive care unit	614	16.3
	Internal medicine	562	14.9
	Surgery	554	14.7
	Outpatient department	294	7.8
	Accident and emergency	293	7.8
	Obstetrics and gynecology	183	4.8
	Neonatology	158	4.2

Data are presented as frequencies and percentages.

### Overall prevalence of ESKAPE pathogens over the five years

**Figure 1:**
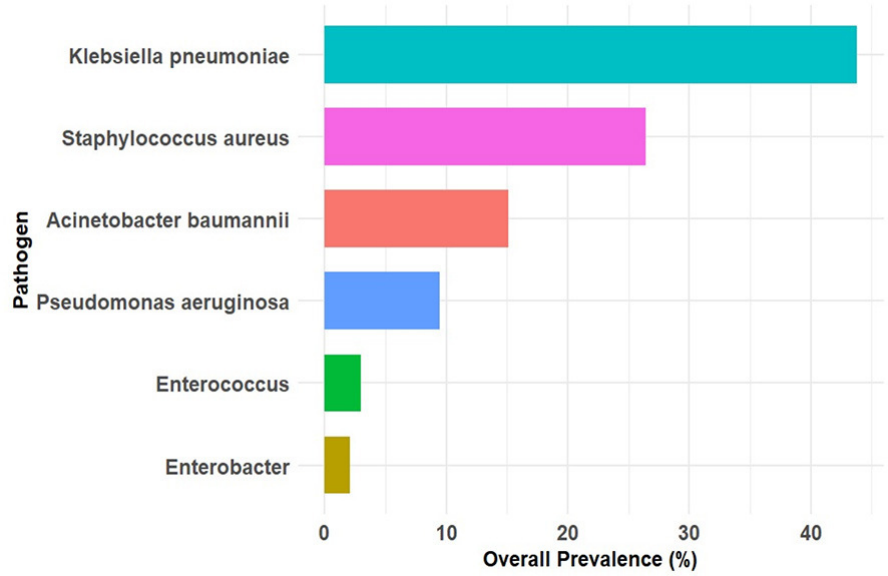
The prevalence of ESKAPE pathogens over the five years. Data are presented as percentages.

*The total isolates were 3,778, among them K. pneumoniae* was the most prevalent pathogen, accounting for 1,655 isolates (43.8%), followed *by S. aureus* (*n* = 999; 26.4%), *A. baumannii* (*n* = 571; 15.1%), and *P. aeruginosa* (*n* = 359; 9.5%). *Enterococcus spp*. (*n* = 113; 3%) and *Enterobacter spp*. (*n* = 81; 2.1%) were comparatively infrequent (Figure 1).

### Annual prevalence of each pathogen among clinical samples

**Figure 2:**
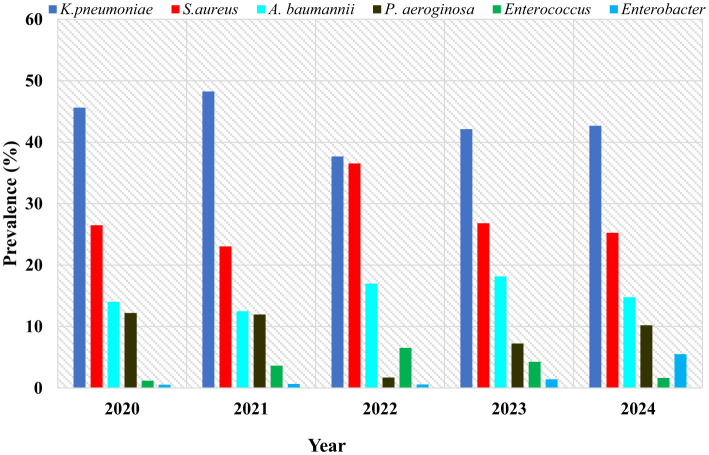
Prevalence of each pathogen per year among collected clinical samples. Data are presented as percentages.

*K. pneumoniae* consistently dominated across all the years, ranging from 37.0% to 48.2%, peaking in 2021. The prevalence of *S. aureus* was highest in 2022 (36.5%) but lowest in 2021 (23.0%). *P. aeruginosa* prevalence was at its highest in 2020 (12.2%) but declined sharply in 2022 (1.7%), followed by partial recovery in subsequent years to 10.2% in 2024, this fluctuation may reflect changes in specimen distribution, ICU admission patterns, and variations in diagnostic sampling practices during that period, rather than a true epidemiological decline to know the true reason for this it requires inferential statistics. Despite a slight decline in 2021 (12.5%), *A. baumannii* gradually increased from 14.0% in 2020 to 18.2% in 2023 before declining again in 2024. *Enterobacter spp*. rose to 5.5% in 2024, compared with consistently low levels in previous years, indicating an emerging trend. Despite being consistently low during the study period, *Enterococcus spp*. peaked in 2022 (6.5%) and gradually declined again, registering 1.6% in 2024 (Figure 2).

### ESKAPE pathogens by sample type

**Figure 3:**
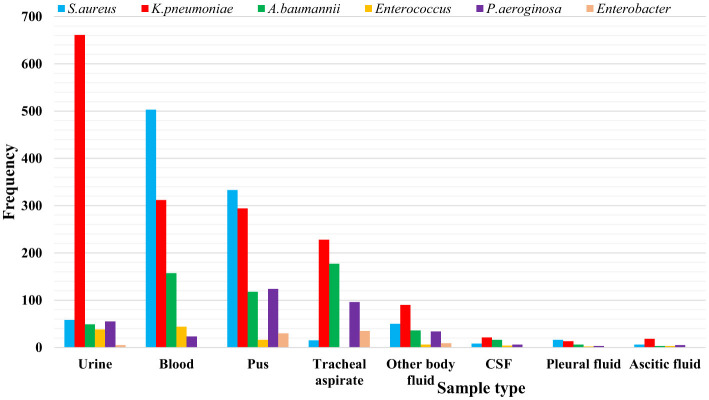
Distribution of ESKAPE pathogens by sample type. Data are presented as frequency (counts). CSF, Cerebrospinal fluid.

*K. pneumoniae* was predominantly isolated from urine (*n* = 661) and blood (*n* = 312) samples and was also recovered from tracheal aspirates (*n* = 228) and pus (*n* = 294). *S. aureus* was most frequently recovered from blood (*n* = 503) and pus (*n* = 333). *A. baumannii* and *P. aeruginosa* were primarily isolated from tracheal aspirates (*n* = 177 and *n* = 96, respectively). *Enterococcus species* were mainly detected in blood (*n* = 44) and urine (*n* = 38), while *Enterobacter spp*., although infrequent, were recovered from tracheal aspirates (*n* = 35) and pus (*n* = 30) (Figure 3).

### Distribution of ESKAPE pathogens by demographic characteristics

The distribution of ESKAPE pathogens varied significantly across age groups, with the highest burden observed among individuals of reproductive age (*p* < 0.001). *S. aureus* was more common among children under five and pediatric patients, while *Enterococcus spp*. were more frequently identified among the older adults. Significant differences were also observed across hospital departments (*p* < 0.001). The ICU accounted for a substantial proportion of *A. baumannii* and *P. aeruginosa* isolates. Moreover, pediatric wards contributed the highest proportion of *S. aureus, K. pneumoniae*, and *Enterobacter spp*.

Pathogen distribution further varied by specimen type (*p* < 0.001). *S. aureus* was predominantly isolated from blood samples, while *K. pneumoniae* was most frequently recovered from urine. *A. baumannii* and *P. aeruginosa* were mainly isolated from tracheal aspirates, whereas pus samples contributed substantially to *Enterobacter spp*. and *P. aeruginosa* isolates. In addition, a significant temporal variation was observed across the study period (*p* < 0.001). *K. pneumoniae* remained consistently prevalent throughout all years, while *Enterobacter spp*. showed a marked increase in 2024, and fluctuations were observed in *A. baumannii* and *P. aeruginosa*, indicating dynamic changes in pathogen distribution over time (Table 2).

**Table 2:** Comparison between ESKAPE pathogens and demographic characteristics.

Variables		A. baumannii (%)	Enterobacter (%)	Enterococcus (%)	K. pneumoniae (%)	P. aeruginosa (%)	S. aureus (%)	*P*-value
Age category	Neonates	12 (2.1%)	1 (1.2%)	1 (0.9%)	49 (3%)	5 (1.4%)	90 (9%)	< 0.001
	Under five	71 (12.4%)	15 (18.5%)	6 (5.3%)	269 (16.3%)	52 (14.5%)	243 (24.3%)	
	Pediatrics	42 (7.4%)	15 (18.5%)	6 (5.3%)	163 (9.8%)	59 (16.4%)	182 (18.2%)	
	Reproductive age	221 (38.7%)	29 (35.8%)	39 (34.5%)	613 (37%)	133 (37%)	316 (31.6%)	
	Adults	82 (14.4%)	9 (11.1%)	20 (17.7%)	197 (11.9%)	45 (12.5%)	91 (9.1%)	
	Elders	143 (25%)	12 (14.8%)	41(36.3%)	364 (22%)	65(18.1%)	77 (7.7%)	
Gender	Female	252 (44.1%)	35 (43.2%)	57 (50.4%)	802 (48.5%)	146 (40.7%)	459 (45.9%)	0.078
	Male	319 (55.9%)	46 (56.8%)	56 (49.6%)	853 (51.5%)	213 (59.3%)	540 (54.1%)	
Period	2020	107 (18.7%)	4 (4.9%)	9 (8%)	348 (21%)	93 (25.9%)	202 (20.2)	< 0.001
	2021	96 (16.8%)	5 (6.2%)	28 (24.8%)	371 (22.4%)	92 (25.6%)	177 (17.7%)	
	2022	60 (10.5%)	2 (2.5%)	23 (20.4%)	133 (8%)	6 (1.7%)	129 (12.9)	
	2023	153 (26.8%)	12 (14.8%)	36 (31.9%)	355 (21.5%)	61 (17%)	226 (22.6)	
	2024	155 (27.1%)	58 (71.6%)	17 (15%)	448 (27.1%)	107 (29.8%)	265 (26.5%)	
Department	A&E	43 (7.5%)	2 (2.5%)	20 (17.7%)	127 (7.7%)	13 (3.6%)	88 (8.8)	*p* < 0.001
	ICU	169 (29.6%)	16 (19.8%)	19 (16.8%)	254 (15.3%)	89 (24.8%)	67 (6.7%)	
	IM	109 (19.1%)	10 (12.3%)	27 (23.9%)	265 (16%)	41 (11.4%)	110 (11%)	
	Neonatology	12 (2.1%)	1 (1.2%)	1 (0.9%)	49 (3%)	5 (1.4%)	90 (9%)	
	Obs&gyn	21 (3.7%)	1 (1.2%)	10 (8.8%)	95 (5.7%)	9 (2.5%)	47 (4.7%)	
	OPD	24 (4.2%)	6 (7.4%)	14 (12.4%)	183 (11.1%)	19 (5.3%)	48 (4.8%)	
	Pediatrics	113 (19.8%)	30 (37%)	12 (10.6%)	432 (26.1%)	111 (30.9%)	422 (42.2%)	
	Surgery	80 (14%)	15 (18.5%)	10 (8.8%)	250 (15.1%)	72 (20.1%)	127 (12.7%)	
Sample type	Ascitic fluid	3(0.5%)	0 (0%)	3 (2.7%)	18 (1.1%)	5 (1.4%)	6 (0.6%)	*p* < 0.001
	Blood	157 (27.5%)	0 (0%)	44 (38.9%)	312 (18.9%)	23 (6.4%)	503 (50.4%)	
	CSF	16 (2.8%)	0 (0%)	4 (3.5%)	21 (1.3%)	6 (1.7%)	8 (0.8%)	
	Other body fluid	36 (6.3%)	9 (11.1%)	6 (5.3%)	90 (5.4%)	34 (9.5%)	50 (5%)	
	Pleural fluid	6 (1.1%)	0 (0%)	2 (1.8%)	13 (0.8%)	3 (0.8%)	16 (1.6%)	
	Pus	118 (20.7%)	30 (37%)	16 (14.2%)	294 (17.8%)	124 (34.5%)	333 (33.3%)	
	Tracheal aspirate	177 (31%)	35 (43.2%)	0 (0%)	228 (13.8%)	96 (26.7)	15(1.5%)	
	Urine	49 (8.6%)	5 (6.2%)	38 (33.6%)	661 (39.9%)	55 (15.3%)	58 (5.8%)	
	Wound swab	9 (1.6%)	2 (2.5%)	0 (0%)	18 (1.1%)	13 (3.6%)	10 (1%)	

Data are presented as frequencies and percentages (%). The age categories were classified as: Neonates (0–28 days), Under five (1–59 months), Pediatrics (5–18 years), Reproductive age (19–45 years), Adults (46–60 years), and Elders (>60 years). The significance level of the difference between categorical variables was tested using the Chi-square test or Fisher's exact test, and a *P* value less than 0.05 was considered statistically significant.

### Overall antimicrobial resistance rate among ESKAPE pathogens

The overall resistance rate among tested pathogens was 56.5% *with Enterococcus spp*. exhibited the highest resistance rate (557/747; 74.6%), followed by *A. baumannii* (2,759/3,946; 69.9%) and *Enterobacter* spp. (382/593; 64.4%). *P. aeruginosa* showed a resistance rate of 1,472/2,405 (61.2%). *K. pneumoniae* and S. *aureus* demonstrated lower resistance rates among the pathogens, registering 7,532/12,776 (59.0%) and 3,316/7,886 (42.0%), respectively. Overall, across all 28,353 antimicrobial susceptibility tests performed, 16,018 (56.5%) were resistant, indicating a substantial burden of antimicrobial resistance (Table 3).

**Table 3:** Antimicrobial resistance rate among ESKAPE pathogens.

Pathogen	Total tests	Resistant *n* (%)
*Enterococcus*	747	557 (74.6)
*Acinetobacter baumannii*	3,946	2,759 (69.9)
*Enterobacter*	593	382 (64.4)
*Pseudomonas aeruginosa*	2,405	1,472 (61.2)
*Klebsiella pneumoniae*	12,776	7,532 (59.0)
*Staphylococcus aureus*	7,886	3,316 (42.0)
**Overall**	**28,353**	**16,018 (56.5)**

Data are presented as frequencies and percentages. Bold indicate Overall/Total.

### Resistance trend per the WHO AWaRe classification over the five years

**Figure 4:**
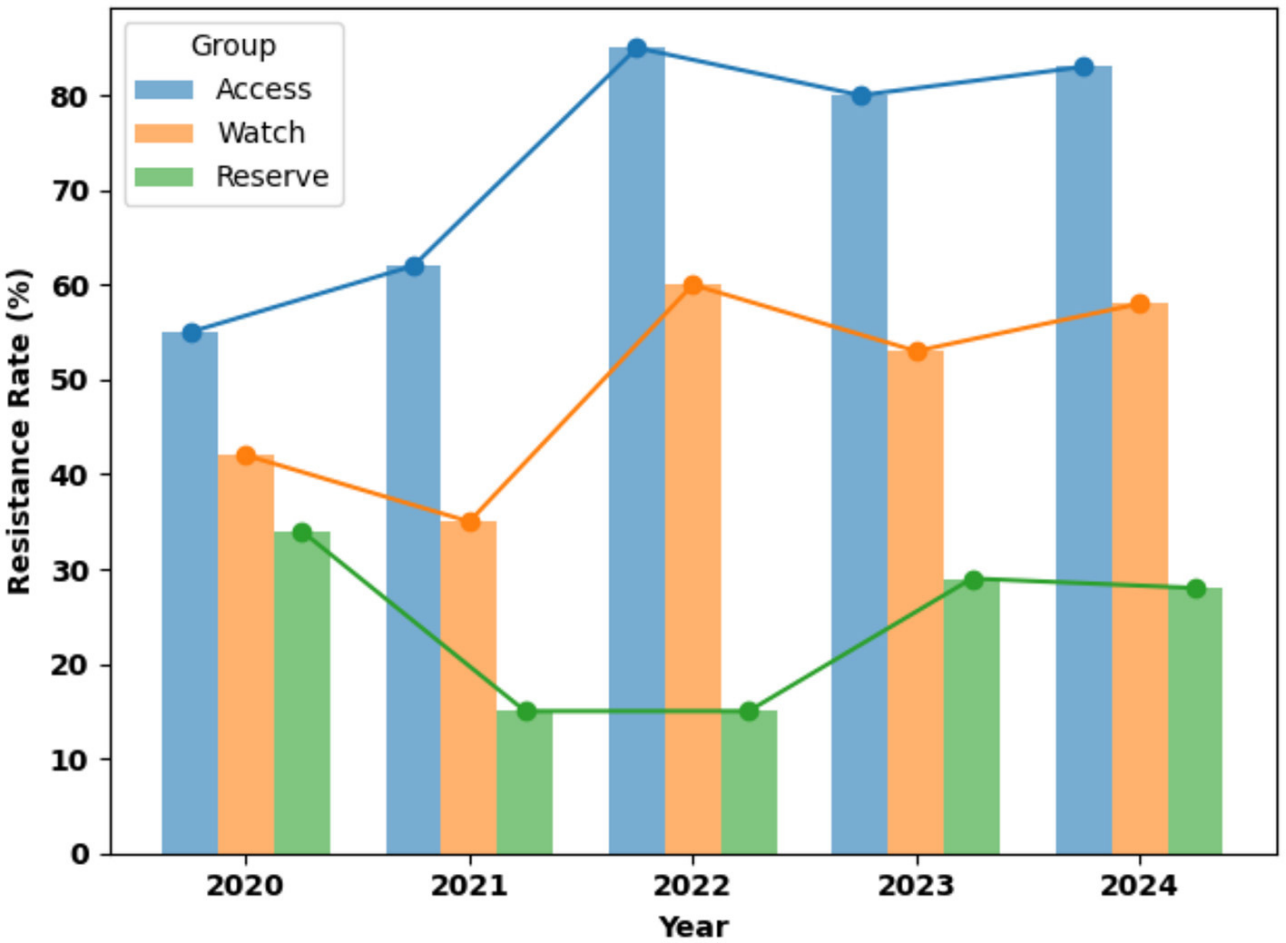
Resistance trend per WHO AWaRe antibiotic classification over the five years. Data are presented as percentages.

Figure 4 illustrates persistently high and generally increasing resistance rates in the Access and Watch AWaRe categories between 2020 and 2024. Resistance to access antibiotic category was already substantial in 2020, approximately 55%, and rose sharply thereafter, peaking in 2022 at around 86% and remaining above 80% through 2024. The watch antibiotic category exhibited moderate resistance with noticeable fluctuations, declining from about 43% in 2020 to 35% in 2021, followed by a marked increase to approximately 60% in 2022 and sustained levels above 50% in subsequent years. In contrast, Reserve antibiotics consistently showed the lowest resistance rates, with a marked decrease from about 35% in 2020 to around 15% in 2021 and 2022, followed by a modest increase to approximately 28–29% in 2023 and 2024. The average resistance rate in the last 5 years was 79.8% for Access antibiotics, 51.6% for Watch antibiotics, and 24.4% for Reserve antibiotics. Overall, the figure highlights a worrying burden of resistance among first- and second-line antibiotics, emphasizing challenges for empirical therapy and the critical need for strengthened antimicrobial stewardship and infection prevention efforts.

### Prevalence of common resistance phenotypes

**Figure 5:**
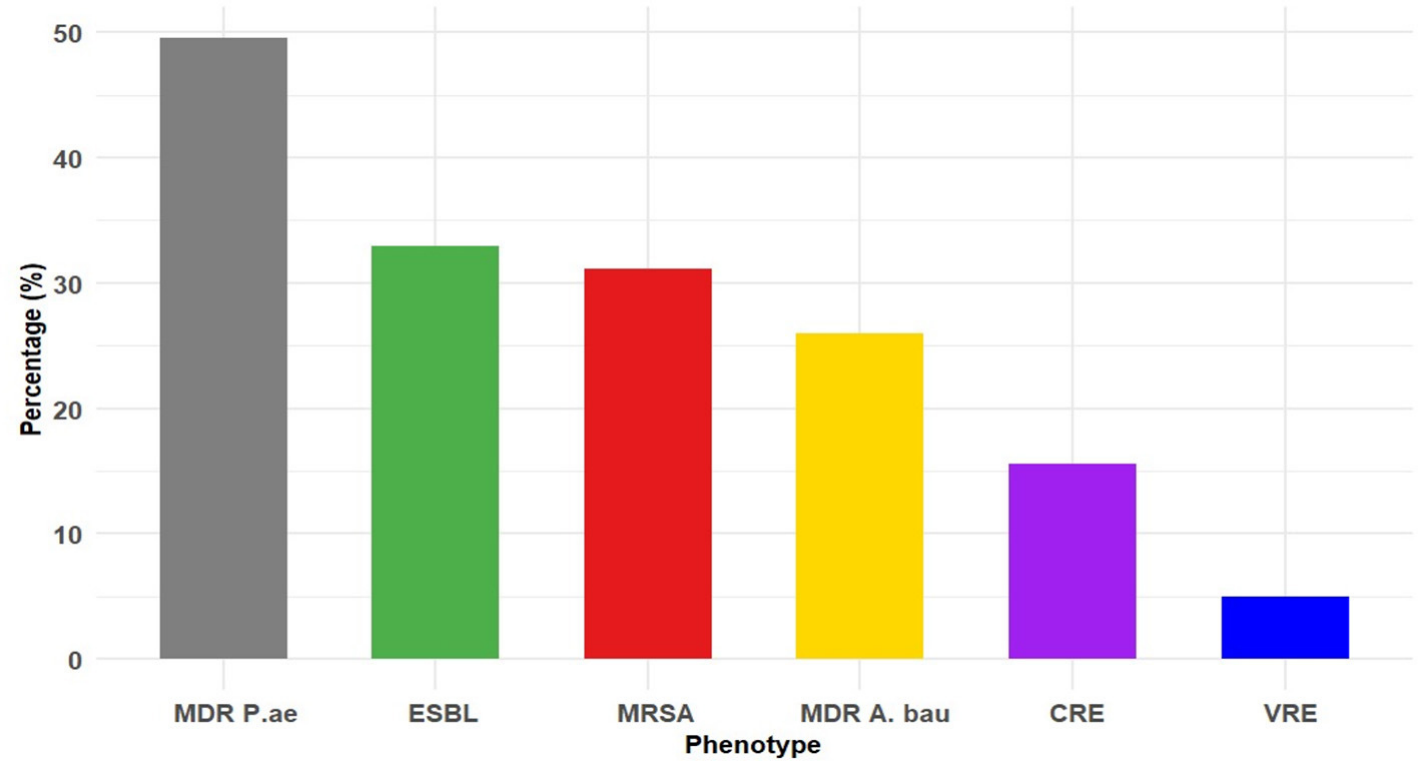
Prevalence of the resistance phenotypes. Data are presented as percentages. ESBL, extende spectram beta lactamase; MDR P.ae, multidrug-resistant *Pseudomonas aeruginosa*; MRSA, methicillin-resistant *Staphylococcus aureus*; MDR A.bau, multidrug-resistant *Acinetobacter baumanii*; CRE, carbapenem-resistant Enterobacterale; VRE, vancomycin-resistant *Enterococci*.

Figure 5 demonstrates a high burden of antimicrobial resistance among the tested isolates. Multidrug-resistant *Pseudomonas* was most prevalent, detected with 141 of 285 isolates (49.5%), followed by ESBL-producing organisms, 423 of 1,286 isolates (32.9%). Methicillin-resistant *Staphylococcus aureus* (MRSA) accounted for 237 of 763 isolates (31.1%). Multidrug-resistant *Acinetobacter* was found in 124 of 477 isolates (26%). Carbapenem-resistant *Enterobacterale* (CRE) were identified in 200 of 1,286 isolates (15.6%), and vancomycin-resistant Enterococci (VRE) were the least common, occurring in 4 of 80 isolates (5%) (Figure 5).

### Antibiotic resistance of gram-positive ESKAPE pathogens

Table 5 indicates a substantial burden of antimicrobial resistance among Gram-positive ESKAPE pathogens, particularly *Enterococcus spp*. and *Staphylococcus aureus*. *Enterococcus spp*. demonstrated very high resistance to most antibiotics tested, including ampicillin 24/29 (82.76%), clindamycin 83/94 (88.30%), erythromycin 91/102 (89.22%), tetracycline 77/85 (90.59%), cotrimoxazole 13/15 (86.67%), and penicillin G 99/103 (96.12%), reflecting markedly limited treatment options. Resistance to vancomycin among *Enterococcus spp*. was relatively low, 9/113 (7.97%), suggesting retained activity of this last-line agent.

*Staphylococcus aureus* exhibited very high resistance to penicillin G 867/928 (93.43%), while moderate resistance was observed to ciprofloxacin 114/294 (38.78%), gentamicin 43/100 (43.0%), clindamycin 312/894 (34.90%), erythromycin 409/959 (42.65%), tetracycline 363/831 (43.68%), and cotrimoxazole 102/201 (50.75%). Resistance to oxacillin 296/833 (35.53%) and cefoxitin 244/563 (43.34%), indicative of methicillin resistance, suggests a considerable prevalence of MRSA. Vancomycin resistance in *S. aureus* remained rare, 10/962 (0.8%), supporting its continued effectiveness for severe infections. Overall, these findings underscore extensive resistance to commonly used antibiotics among Gram-positive pathogens and highlight the critical need for susceptibility-guided therapy and strengthened antimicrobial stewardship (Table 4).

**Table 4:** Antibiotic resistance in gram-positive ESKAPE pathogens.

Antibiotic	*Enterococcus*	*S. aureus*
Ampicillin	24/29 (82.76%)	234/246 (95.12%)
Chloramphenicol	24/35 (68.57%)	64/360 (17.78%)
Ciprofloxacin	19/26 (73.08%)	114/294 (38.78%)
Gentamicin	10/15 (66.67%)	43/100 (43%)
Clindamycin	83/94 (88.30%)	312/894 (34.90%)
Erythromycin	91/102 (89.22%)	409/959 (42.65%)
Nitrofurantoin	1/2 (50%)	3/6 (50%)
Cefoxitin	7/7 (100%)	244/563 (43.34%)
Oxacillin	30/31 (96.7%)	296/833 (35.53%)
Penicillin G	99/103 (96.12%)	867/928 (93.43%)
Cotrimoxazole	13/15 (86.67%)	102/201 (50.75%)
Tetracycline	77/85 (90.59%)	363/831 (43.68%)
Vancomycin	9/113 (7.965%)	10/962 (0.8%)

Data are presented as percentages of resistant isolates.

### Antibiotic resistance in gram-negative ESKAPE pathogens

Table 5 a high level of antimicrobial resistance among Gram-negative ESKAPE pathogens, including *Acinetobacter spp*., *Enterobacter spp*., *Klebsiella pneumoniae*, and *Pseudomonas aeruginosa*. *Acinetobacter spp*. exhibited extensive resistance to multiple antibiotic classes, notably amoxicillin–clavulanate 307/311 (98.71%), third- and fourth-generation cephalosporins such as ceftazidime 387/429 (90.21%), ceftriaxone 167/180 (92.78%), and cefotaxime 188/201 (93.53%), as well as fluoroquinolones and aminoglycosides, including ciprofloxacin 373/464 (80.39%) and gentamicin 327/418 (78.23%); carbapenem resistance was also substantial with imipenem 223/418 (53.35%) and meropenem 95/167 (56.89%). *Enterobacter spp*. demonstrated high resistance to β-lactams, including amoxicillin-clavulanate 68/70 (97.14%) and cephalosporins, ceftazidime 53/63 (84.13%), with lower but notable resistance to carbapenems, particularly imipenem 27/61 (44.26%), while meropenem resistance remained relatively low 1/10 (10%).

**Table 5:** Antibiotic resistance in gram-negative ESKAPE pathogens.

Antibiotic	*Acinetobacter*	*Enterobacter*	*K. pneumoniae*	*P.aeruginosa*
Amikacin	81/430 (18.84%)	6/59 (10.17%)	194/1,237 (15.68%)	73/262 (27.86%)
Amoxicillin/ Clavulanate	307/311 (98.71%)	68/70 (97.14%)	1,427/1,542 (92.54%)	143/143 (100%)
Ceftazidime	387/429 (90.21%)	53/63 (84.13%)	914/1,147 (79.69%)	199/300 (66.33%)
Cefepime	53/68 (77.94%)	9/13 (69.23%)	116/152 (76.32%)	26/41 (63.41%)
Ciprofloxacin	373/464 (80.39%)	47/69 (68.12%)	709/1,301 (54.5%)	179/302 (59.27%)
Gentamicin	327/418 (78.23%)	44/68 (64.71%)	860/1,202 (71.55%)	197/273 (72.16%)
Ceftriaxone	167/180 (92.78%)	30/36 (83.33%)	848/1,051 (80.69%)	88/93 (94.62%)
Cefotaxime	188/201 (93.53%)	25/30 (83.33%)	796/999 (79.68%)	102/109 (93.58%)
Imipenem	223/418 (53.35%)	27/61 (44.26%)	203/1,253 (16.20%)	128/266 (48. 1%)
Meropenem	95/167 (56.89%)	1/10 (10%)	123/613 (20.07%)	55/115 (47.83%)
Polymyxin B	44/209 (21.05%)	8 (42.11%)	164/492 (33.33%)	20/128 (15.63%)
Cotrimoxazole	109/150 (72.67%)	27/21 (87.1%)	458/561 (81.64%)	79/81 (97.53%)
Piperacillin/ Tazobactam	294/376 (78.19%)	30 (58.82%)	361/703 (51.73%)	148/252 (58.73%)

Data are presented as frequencies and percentages (%).

*K. pneumoniae* showed widespread resistance to cephalosporins, ceftriaxone 848/1,051 (80.69%), gentamicin 860/1,202 (71.55%), and cotrimoxazole 458/561 (81.64%), although resistance to carbapenems was comparatively lower for imipenem 203/1,253 (16.20%) and meropenem 123/613 (20.07%). *P. aeruginosa* displayed particularly high resistance to amoxicillin–clavulanate 143/143 (100%), extended-spectrum cephalosporins ceftriaxone 88/93 (94.62%); cefotaxime 102/109 (93.58%), and cotrimoxazole 79/81 (97.53%), with moderate resistance to carbapenems imipenem 128/266 (48.12%); meropenem 55/115 (47.83%). Across all organisms, polymyxin B resistance remained lower than most other agents but was still notable, especially in *K. pneumoniae* 164/492 (33.33%). Overall, these findings highlight widespread multidrug resistance among Gram-negative pathogens, severely limiting treatment options and emphasizing the urgent need for strengthened antimicrobial stewardship and infection control measures (Table 5).

## Discussion

This study presents a comprehensive analysis of the prevalence and AMR profiles of ESKAPE pathogens at CHUK, the largest tertiary referral hospital in Rwanda, providing important insight into local epidemiological and resistance trends. The most prevalent pathogens were *K. pneumoniae* (43.8%), *S. aureus* (26.4%), and *A. baumannii* (15.1%), with an overall resistance rate exceeding 50%. This distribution is consistent with global patterns, indicating that ESKAPE pathogens are major contributors to antimicrobial resistance (4, 12). These organisms are widely recognized as priority pathogens because of their ability to evade antimicrobial therapy and cause severe, difficult-to-treat infections (4, 13). The observed increase in resistance, particularly to WHO Access and Watch antibiotics, reflects broader global trends driven by antibiotic misuse, limited stewardship, and strong selective pressure in hospital environments (14, 15). Collectively, these findings highlight an evolving AMR landscape that threatens the effectiveness of standard empirical treatment regimens, particularly in LMICs.

*K. pneumoniae* was the most frequently isolated ESKAPE pathogen throughout the study period, accounting for more than 40% of all isolates. Its predominance in urine and bloodstream specimens underscores its central role in urinary tract infections and sepsis, conditions often associated with prolonged hospitalization and invasive procedures. Similar dominance of *K. pneumoniae* has been reported in other hospital-based studies, reflecting its adaptability, environmental persistence, and their involvement in healthcare-associated infection (4, 16).

*S. aureus* was the second most prevalent pathogen, with a notable peak in 2022. Its strong association with bloodstream and wound infections is consistent with its well-established role in invasive hospital-acquired infections and associated mortality (17). The increasing presence of *A. baumannii*, particularly in tracheal aspirates, highlights its growing importance in ventilator-associated pneumonia in the ICU. This observation aligns with its recognized role in severe hospital-acquired infections and its classification by WHO as a critical-priority pathogen (13). The increase in *Enterobacter spp*. observed in 2024, although numerically modest, is epidemiologically important because of the organism's inducible β-lactam resistance and potential to cause outbreaks in healthcare settings (4). Similar pathogen distributions have been reported in other tertiary hospitals in Rwanda, suggesting a consistent national pattern (18). The high frequency and predominance of these pathogens could be associated with poor IPC practices, which could favor HAIs, especially in critical areas such as the ICU.

The study revealed a high overall antimicrobial resistance rate of 56.5% among all tested isolates. *Enterococcus spp*. exhibited the highest resistance rate (74.6%), followed by *A. baumannii* (69.9%) and *Enterobacter spp*. (64.4%), highlighting the urgent need for targeted antimicrobial stewardship interventions (5, 19). In contrast, *S. aureus* demonstrated comparatively lower resistance rates (42%), a pattern also reported in other LMIC settings (20). The lower resistance observed in *S. aureus* compared with Gram-negative bacteria may reflect differences in resistance mechanisms, antibiotic exposure patterns, and infection control practices. Nevertheless, the presence of MRSA remains clinically significant and requires continued surveillance (4, 16). High resistance among ESKAPE pathogens has consistently been associated with increased mortality, prolonged hospital stays, and higher healthcare costs (17, 21).

Resistance trends based on the WHO AWaRe classification revealed an alarming increase in resistance to Access antibiotics, rising from 54.7 % in 2020 to 83.3 % in 2024, indicating a substantial decline in the effectiveness of first-line treatments (1). This finding is particularly concerning because Access antibiotics are intended to be widely available, affordable, and effective for the treatment of common infections. Similar increases in resistance to Access agents have been linked to irrational prescribing practices and limited antimicrobial stewardship oversight in LMICs (15, 22).

Resistance among Watch antibiotics also remained high, increasing from 42.5 % in 2020 to 57.2 % in 2024. At the same time, emerging resistance to Reserve antibiotics (15.3 % in 2022 and 27.7 % in later years) represents a worrying development that threatens the effectiveness of last-resort therapies for multidrug-resistant infections (14, 15). These trends emphasize the urgent need for strengthened IPC measures and robust antimicrobial stewardship programs (7). Comparable patterns have been reported in South Asia and China, where excessive antibiotic use in hospital settings has contributed to rising resistance levels (23, 24).

The study also identified a high prevalence of ESBL producers (32.9 %) and MRSA (31.1 %), findings consistent with previous reports (25, 26). These results are also aligned with global evidence of persistent MRSA transmission in healthcare settings (4, 16). The high prevalence of ESBL-producing organisms significantly reduces the effectiveness of third-generation cephalosporins and increases reliance on carbapenems. Although CRE (15.6%) and VRE (5 %) were less common, they remain epidemiologically important due to their potential for rapid dissemination and the limited treatment options available, underscoring the need for early containment strategies (27, 28).

Antibiotic susceptibility profiles indicated widespread multidrug resistance among both Gram-positive and Gram-negative ESKAPE pathogens. High resistance rates were observed for beta-lactams, fluoroquinolones, and aminoglycosides, with notable carbapenem resistance among *K. pneumoniae* isolates, raising concerns about the diminishing effectiveness of these critical antibiotics (5). In contrast, reserve antibiotics such as ceftazidime-avibactam, polymyxin B, daptomycin, and teicoplanin retained high activity, highlighting their importance in the management of multidrug-resistant infections (28).

These findings reflect broader challenges faced by LMICs, including limited diagnostic capacity, reliance on empirical treatment, and insufficient antimicrobial surveillance systems, all of which contribute to the growing AMR crisis (6, 7, 29). The specimen-specific distribution of pathogens observed in this study provides valuable insights for clinical management and infection control prioritization at CHUK, supporting the need for locally tailored and evidence-based interventions.

This study has several limitations. First, the retrospective design relied on routine laboratory records, which limited control over missing data and changes in testing practices and prevented causal inference between antibiotic use and resistance patterns. Second, as a single-center study conducted at a tertiary referral hospital (CHUK), the findings may not be generalizable to other healthcare settings and may overestimate resistance due to referral bias. Third, the analysis was limited to phenotypic susceptibility testing without molecular characterization, preventing identification of specific resistance mechanisms and transmission pathways. Additionally, the absence of patient-level clinical data and antibiotic consumption information limited the ability to assess risk factors, clinical outcomes, and the relationship between prescribing practices and resistance trends. Finally, small sample sizes for some pathogens and specimen types in certain years may have affected the stability of trend analyses and contributed to fluctuations in yearly prevalence estimates.

## Conclusion

This study demonstrates a high prevalence of multidrug-resistant ESKAPE pathogens, with *K. pneumoniae, S. aureus*, and *A. baumannii* emerging as leading pathogens. The escalating resistance to access and watch antimicrobials, alongside emerging resistance to reserve agents, highlights an urgent need to strengthen antimicrobial stewardship programs and infection prevention and control measures. Enhancing laboratory capacity for routine and fast antimicrobial susceptibility testing and promoting evidence-based antibiotic prescribing are critical to mitigating the AMR burden. While reserve antibiotics largely retained efficacy, cautious and judicious use is essential to preserve their therapeutic value. Overall, this study provides essential local epidemiological data to inform national AMR control strategies in Rwanda and supports the implementation of a One Health approach to comprehensively address antimicrobial resistance through continuous surveillance.

## Data Availability

The original contributions presented in the study are included in the article/Supplementary material, further inquiries can be directed to the corresponding author.

## References

[1] World Health Organization. Global Antimicrobial Resistance and Use Surveillance System (GLASS). Geneva: WHO (2021). Available online at: https://www.who.int/initiatives/glass (Accessed December 16, 2025).

[2] Naghavi M Vollset SE Ikuta KS Swetschinski LR Gray AP Wool EE . Global burden of bacterial antimicrobial resistance 1990–2021: a systematic analysis with forecasts to 2050. Lancet. (2024) 404:1199–226. doi: 10.1016/S0140-6736(24)01867-1 39299261 PMC11718157

[3] Murray CJL Ikuta KS Sharara F Swetschinski L Aguilar GR Gray A . Global burden of bacterial antimicrobial resistance in 2019: a systematic analysis. Lancet. (2022) 399:629–55. 35065702 10.1016/S0140-6736(21)02724-0 PMC8841637

[4] De Oliveira DMP Forde BM Kidd TJ Harris PNA Schembri MA Beatson SA. et al. Antimicrobial resistance in ESKAPE pathogens. Clin Microbiol Rev. (2020) 33:e00181-19. doi: 10.1128/CMR.00181-19 PMC7227449 32404435

[5] Ayobami O Brinkwirth S Eckmanns T Markwart R. Antibiotic resistance in hospital-acquired ESKAPE-E infections in low- and lower-middle-income countries: a systematic review and meta-analysis. Emerg Microbes Infect. (2022) 11:443–51. doi: 10.1080/22221751.2022.2030196 35034585 PMC8820817

[6] Tadesse BT Ashley EA Ongarello S Havumaki J Wijegoonewardena M Gonzalez IJ . Antimicrobial resistance in Africa: a systematic review. BMC Infect Dis. (2017) 17:616. doi: 10.1186/s12879-017-2713-1 28893183 PMC5594539

[7] FAO, UNEP, WHO, WOAH, One One Health Joint Plan of Action (2022–2026). Working Together for the Health of Humans, Animals, Plants and the Environment. Rome (2022). Available online at: https://www.who.int/publications/i/item/9789240059139 (Accessed December 16, 2025).

[8] AMR Initiative Rwanda, Research & Surveillance, Kigali (2024). Available online at: https://amrinitiativerwanda.org/research-surveillance/ (Accessed January 8, 2026).

[9] Munyemana JB Gatare B Kabanyana P Ivang A Mbarushimana D Itangishaka I . Antimicrobial resistance profile of bacteria causing pediatric infections at the university teaching hospital in Rwanda. Am J Trop Med Hyg. (2022) 107:1308–14. doi: 10.4269/ajtmh.22-0047 36216320 PMC9768258

[10] Habyarimana T Murenzi D Musoni E Yadufashije C Niyonzima FN. Bacteriological profile and antimicrobial susceptibility patterns of bloodstream infection at Kigali university teaching hospital. Infect Drug Resist. (2021) 14:699–707. doi: 10.2147/IDR.S299520 33654414 PMC7914060

[11] Rwanda Rwanda Ministry of Health Ministry Ministry of Agriculture and Animal Resources Ministry Ministry of Environment. National Action Plan on Antimicrobial Resistance (2024). Available online at: https://www.rbc.gov.rw/fileadmin/user_upload/strategy/2nd_NATIONAL_ACTION_PLAN_ON_AMR__NAP_2025-2029_.pdf (Accessed January 5, 2026).

[12] Miller WR Arias CA. ESKAPE pathogens: antimicrobial resistance, epidemiology, clinical impact and therapeutics. Nat Rev Microbiol. (2024) 22:598–616. doi: 10.1038/s41579-024-01054-w 38831030 PMC13147291

[13] Sati H Carrara E Savoldi A Hansen P Garlasco J Campagnaro E . The WHO bacterial priority pathogens list 2024: a prioritisation study to guide research, development, and public health strategies against antimicrobial resistance. Lancet Infect Dis. (2025) 25:1033–43. doi: 10.1016/S1473-3099(25)00118-5 40245910 PMC12367593

[14] Ramzan S Butt AT Khowaja W Ghani S. Prescribing patterns of antibiotics according to the WHO access, watch, and reserve (AWaRe) classification in the pediatric outpatient department: a prospective study. Pak J Med Sci. (2025) 41:3169–76. doi: 10.12669/pjms.41.11.12169 41394358 PMC12697030

[15] Anand G Lahariya R Priyadarshi K Sarfraz A. From access to reserve: antimicrobial resistance among etiological agents of central line-associated bloodstream infections in the view of WHO's AWaRe antimicrobial spectrum. GMS Hyg Infect Control. (2025) 20:Doc30. doi: 10.3205/dgkh000559 40657631 PMC12248246

[16] Orhan Z Kirisci O Doganer A Altun M Kucuk B Aral M. Antibiotic resistance trends in ESKAPE pathogens isolated at a health practice and research hospital: a five-year retrospective study. J Infect Dev Ctries. (2024) 18:1899–908. doi: 10.3855/jidc.19592 39832249

[17] Marturano JE Lowery TJ. ESKAPE pathogens in bloodstream infections are associated with higher cost and mortality but can be predicted using diagnoses upon admission. Open Forum Infect Dis. (2019) 6:ofz503. doi: 10.1093/ofid/ofz503 31844639 PMC6902016

[18] Muhinda C Murenzi G Al-Hassan L Seruyange E Mutesa L Gylfe A. High antimicrobial resistance in ESKAPE pathogens at a Rwandan tertiary hospital. Pathogens. (2025) 14:1253. doi: 10.20944/preprints202511.1446.v1 41471208 PMC12736372

[19] Regassa BT Tosisa W Eshetu D Beyene D Abdeta A Negeri AA . Antimicrobial resistance profiles of bacterial isolates from clinical specimens referred to Ethiopian public health institute: analysis of 5-year data. BMC Infect Dis. (2023) 23:798. doi: 10.1186/s12879-023-08803-x 37968587 PMC10647041

[20] Jama-Kmiecik A Maczynska B Frej-Madrzak M Choroszy-Krol I Dudek-Wicher R Piatek D . The changes in the antibiotic resistance of *Staphylococcus aureus, Streptococcus pneumoniae, Enterococcus faecalis* and *Enterococcus faecium* in the clinical isolates of a multiprofile hospital over 6 years (2017–2022). J Clin Med. (2025) 14:332. doi: 10.3390/jcm14020332 39860338 PMC11766039

[21] Ehsan H Ibrahimkhil MA Ahmadi M Wardak FR Aminpoor H Salam A . A cross-sectional evaluation of rational antibiotic prescribing in Kabul based on WHO indicators. BMC Infect Dis. (2025) 25:1385. doi: 10.1186/s12879-025-11826-1 41126089 PMC12542144

[22] Song H Liu X Zou K Li H Fei H Huang L . Assessment of antibiotic consumption patterns in hospital and primary healthcare using WHO access, watch and reserve classification (AWaRe) in Sichuan Western China: 2020. Arch Public Health. (2024) 82:182. doi: 10.1186/s13690-024-01391-5 39402638 PMC11472543

[23] Khatri E Joshi P Banjara MR Dhimal M. Analysis of antibiotics consumption pattern among hospitalized patients in Nepal: a nationally representative multi-hospital survey. BMC Infect Dis. (2025) 25:1655. doi: 10.1186/s12879-025-12187-5 41275124 PMC12648978

[24] Kwok K-O Chan E Chung P-H Tang A Wei W-I Zhu C . Prevalence and associated factors for carriage of Enterobacteriaceae producing ESBLs or carbapenemase and methicillin-resistant *Staphylococcus aureus* in Hong Kong community. J Infect. (2020) 81:242–7. doi: 10.1016/j.jinf.2020.05.033 32447008

[25] Piso RJ Kach R Pop R Zillig D Schibli U Bassetti S . A cross-sectional study of colonization rates with methicillin-resistant *Staphylococcus aureus* (MRSA) and extended-spectrum beta-lactamase (ESBL) and carbapenemase-producing enterobacteriaceae in four Swiss refugee centres. PLoS ONE. (2017) 12:e0170251. doi: 10.1371/journal.pone.0174911 28085966 PMC5234815

[26] Bonomo RA Burd EM Conly J Limbago BM Poirel L Segre JA . Carbapenemase-producing organisms: a global scourge. Clin Infect Dis. (2017) 66:1290–7. doi: 10.1093/cid/cix893 29165604 PMC5884739

[27] Durante-Mangoni E Andini R Zampino R. Management of carbapenem-resistant *Enterobacteriaceae* infections. Clin Microbiol Infect. (2019) 25:943–50. doi: 10.1016/j.cmi.2019.04.013 31004767

[28] Pipitò L Rubino R D'Agati G Bono E Mazzola CV Urso S . Antimicrobial resistance in ESKAPE pathogens: a retrospective epidemiological study at the university hospital of Palermo, Italy. Antibiotics. (2025) 14:186. doi: 10.3390/antibiotics14020186 40001429 PMC11851393

[29] Denissen J Reyneke B Waso-Reyneke M Havenga B Barnard T Khan S . Prevalence of ESKAPE pathogens in the environment: Antibiotic resistance status, community-acquired infection and risk to human health. Int J Hyg Environ Health. (2022) 244:114006. doi: 10.1016/j.ijheh.2022.114006 35841823

