# Supplementary material for: ESKAPE pathogens and their antimicrobial resistance patterns at the university teaching hospital of Kigali, Rwanda: a five -year analysis (2020–2024)
Source: Front Public Health. 2026 Apr 23;14:1783011. doi: 10.3389/fpubh.2026.1783011 (PMC13149477; doi:10.3389/fpubh.2026.1783011)
Supplement: Supplementary file 1 [file Table_1.docx]

**Supplementary Table 1: Antibiotics used for antimicrobial susceptibility testing of ESKAPE pathogens at CHUK (2020–2024)**

| **Antibiotic class** | **Antibiotic** | **Abbreviation** | **Disk content (µg)** | **WHO AWaRe category*** |
| --- | --- | --- | --- | --- |
| **Penicillins** | Ampicillin | AMP | 10 | Access |
|  | Amoxicillin–clavulanic acid | AMC | 20/10 | Access |
|  | Ampicillin–sulbactam | SAM | 10/10 | Access |
|  | Penicillin G^a^ | P | 10 | Access |
| **Cephalosporins** | Cefuroxime | CXM | 30 | Access |
|  | Cefotaxime | CTX | 30 | Watch |
|  | Ceftriaxone | CRO | 30 | Watch |
|  | Ceftazidime | CAZ | 30 | Watch |
|  | Cefoxitin | FOX | 30 | Watch |
|  | Cefoperazone | CFP | 75 | Watch |
| **Carbapenems** | Imipenem | IPM | 10 | Watch |
|  | Meropenem | MEM | 10 | Watch |
| **Aminoglycosides** | Gentamicin | CN | 10 | Access |
|  | Amikacin | AK | 30 | Access |
| **Fluoroquinolones** | Ciprofloxacin | CIP | 5 | Watch |
| **Folate pathway inhibitors** | Trimethoprim–sulfamethoxazole | SXT | 1.25/23.75 | Access |
| **Macrolides** | Erythromycin^a^ | E | 15 | Watch |
| **Lincosamides** | Clindamycin^a^ | DA | 2 | Watch |
| **Tetracyclines** | Tetracycline | TE | 30 | Access |
|  | Doxycycline | DO | 30 | Access |
| **Phenicols** | Chloramphenicol | C | 30 | Access |
| **Glycopeptides** | Vancomycin^b^ | VA | 30 | Watch |
| **Polymyxins** | Polymyxin B | PB | 300 | Reserve |
| **β-lactam/β-lactamase inhibitor** | Piperacillin–tazobactam | TZP | 30/6 | Watch |

* WHO AWaRe classification based on WHO Model List of Essential Medicines

^a^Tested for Gram-positive organisms only

^b^Disk diffusion used according to local SOPs
